# Fatigue Behavior of a Box-Type Welded Structure of Hydraulic Support Used in Coal Mine

**DOI:** 10.3390/ma8105325

**Published:** 2015-09-24

**Authors:** Xiaohui Zhao, Fuyong Li, Yu Liu, Yanjun Fan

**Affiliations:** 1 Key Laboratory of Automobile Materials, School of Materials Science and Engineering, Jilin University, Changchun 130025, China; zhaoxiaohui@jlu.edu.cn (X.Z.); fanyanjunjlu@gmail.com (Y.F.); 2 Zhengzhou Coal Mining Machinery Group Company Limited, Zhengzhou 450016, China; lfyzmj@gmail.com; 3 School of Mechanical Science and Engineering, Jilin University, Changchun 130025, China

**Keywords:** hydraulic support, finite element, stress concentration, weld, fatigue

## Abstract

Hydraulic support is the main supporting equipment of the coal mining systems, and they are usually subjected to fatigue failure under the high dynamic load. The fracture positions are generally at welded joints where there is a serious stress concentration. In order to investigate and further improve the fatigue strength of hydraulic support, the present work first located the possible position where fatigue failure occurs through finite element analysis, and then fatigue tests were carried out on the different forms of welded joints for the dangerous parts. Finally, Fatigue strength-life (*S*-*N*) curves and fracture mechanism were studied. This research will provide a theoretical reference for the fatigue design of welded structures for hydraulic support.

## 1. Introduction

Coal represents the main energy source in the whole world. How to safely and efficiently exploit coal is a key issue [1]. As the main supporting equipment of the coal mining systems, hydraulic support is used to sustain the pressure of coal seams, which is the basic structure to ensure the safety of mining work. With the rapid development of mining technology as well as the large demand of raw coal, hydraulic support is developing towards the high-end and super high-end direction. Hydraulic support contains some box-type structures with a lot of stiffener plates. Welding is the main method to connect these plates into box-type structures. As is known, hydraulic support works under very large dynamic load, which might lead to the occurrence of fatigue failure. Moreover, fatigue failure is sensitive to structural stress concentration. Therefore, it will be worse if the structural stress concentration is located in welded joints. The reason is that welded joint is the weak section in the whole support due to the existence of welded defects. The fatigue performance of welded joint will significantly reduce under the action of structural stress concentration and welded defects.

At present, the service life of the domestic high-end hydraulic support is about 40,000 cycles under actual working load. According to the practical requirements, the service life of the domestic high-end hydraulic support should reach up to 80,000–100,000 cycles in the near future. Structural stress concentration and welded defects as the main factors to influence the fatigue life of hydraulic support, which should be paid high attention during the fracture and fatigue-related failure assessments of welded joints of hydraulic support [2,3]. Therefore, the fatigue performance of welded joints has to be considered when designing the fatigue strength of welded structures [4,5,6,7].

Stress concentration as the main factor affecting the fatigue performance of welded structure has to been considered. Huo and Masubuchi have been studying the assessment of fracture and fatigue-related failure [4,8]. Other scientists have also conducted a lot of research about the influence of stress concentration on the fatigue performance of welded structure. In addition to experimental studies, numerical simulations were also used to thoroughly analyze the stress concentration in welded structures [9,10]. Therefore, taking certain domestic hydraulic supports for example, this paper aimed at investigating and further improving the fatigue strength of hydraulic supports. The major effort first located the position that fatigue failure most likely to happen through finite element analysis, and then carried out fatigue tests for these dangerous parts, followed by studying the fatigue *S*-*N* curves and fracture mechanism. Taking the joints with low fatigue strength into consideration, the forms of welded joints were accordingly optimized. The current research as a combination of finite element simulation and experimental analysis will provide a theoretical reference for the fatigue design of hydraulic support.

## 2. Material and Experimental Procedures

### 2.1. Numerical Modeling

Hydraulic support in this study was shield-type, which consists of the base, connecting rods, shield beams, top beams, pillars, jacks and control facilities. Considering the complex structure of hydraulic supports, the geometrical model was built by three-dimensional modeling software, and then geometrical model was imported into the finite element analysis software for further computation. Base parts were set up first, which was regarded as the design basis. In order to successfully generate mesh and save computing time, the geometrical model was reasonably simplified on the premise that stress state of the main structures was not obviously affected. The principles were as follows [11]: (a) The location of the axes of each part remained unchanged, and the sizes of principal bearing parts were consistent with those in the original design; (b) the details of dangerous parts were retained; (c) the tiny process structures, such as small chamfers and holes that had little influence on stress state were omitted; (d) the equilibrium jack was simplified as a solid pillar, the length of which was determined by the working height of hydraulic support.

Hydraulic support usually bears the supporting load of pillar and the pressure load of working face with surrounding rock. Generally, the blocks are placed in different positions of the support in order to simulate different working conditions in the strength test.

**Figure 1:**
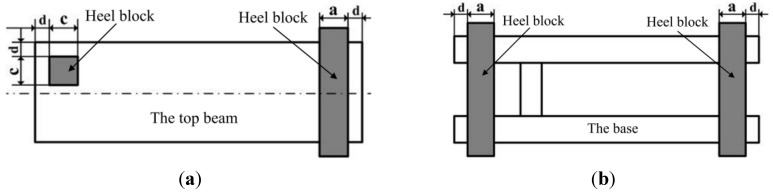
The load is combined by torsion load on the top beam and load on both ends of the base. (**a**) The size of heel block on the top beam (*a* = 150 mm; *c* = 300 mm; *d* = 50 mm; thickness, *h* = 50 mm); (**b**) The size of heel block under the beam (*a* = 150 mm; *d* = 50 mm; thickness, *h* = 50 mm).

In present finite element analysis, one of the most dangerous working condition is concerned, in which the load is combined by torsion load on the top beam and load on both ends of the base [12]. The specific loading form and position are shown in Figure 1.

According to Chinese standards GB-25974.1-2010 [12], the load should be carried out on heel block. However, it is difficult to decide the surface load of heel block based on the nominal working resistance of pillars. Besides, the statically indeterminate problem may occur in finite element analysis. Thus, the pillars were divided into two parts close to the top and bottom nests. When the load is combined by torsion load on the top beam and load on both ends of the base, the height of hydraulic support is calculated according to the following formulas [12].

In this working condition, the minimum height (*H*_min_) of hydraulic support is:

*H*_min_ = 3000 mm (1)

The maximum height (*H*_max_) of hydraulic support is:

*H*_max_ = 4400 mm (2)

The distance (*L*) of hydraulic support is:

*L* = *H*_max_ − *H*_min_ = 4400 mm − 3000 mm = 1400 mm (3)

Therefore, the testing height (*H*) under this working condition is:

*H* = *H*_max_ − *L*/3 = 4400 − 1400/3 mm = 3934 mm (4)

It has been known that if the load of heel block acting on hydraulic support was considered an external load, the heel block-support system will become a statically indeterminate system, which cannot be solved by equilibrium Equations. Therefore, the load of heel block acting on the support was considered as boundary conditions, while the nominal working resistance of pillars was imposed on the cross section of pillars near the base and top beam.

According to Chinese standards GB-25974.1-2010 [12], the rated working resistance (*F*_r_) of single column for this type of hydraulic support is 6000 kN. The actual load (*F*_a_) is 1.2 times of the rated load.

*F*_a_ = 1.2 × *F*_r_ = 1.2 × 6000 kN = 7200 kN (5)

The cross-sectional area of live column and outer cylinder is:

(6) $$S_{1}=\frac{\text{π}d_{1}^{2}}{4}=\frac{\text{π}\cdot 180^{2}}{4}{\text{mm}}^{2}$$

(7) $$S_{2}=\frac{\text{π}d_{2}^{2}}{4}=\frac{\text{π}\cdot 300^{2}}{4}{\text{mm}}^{2}$$

The surface load of live column and outer cylinder is:

(8) $$P_{1}=\frac{F^{\prime }\times 10^{3}}{S_{1}}=\frac{7.2\times 10^{6}}{\frac{\text{π}\cdot 180^{2}}{4}}{\text{mm}}^{2}=283\text{ MPa}$$

(9) $$P_{2}=\frac{F^{\prime }\times 10^{3}}{S_{2}}=\frac{7.2\times 10^{6}}{\frac{\text{π}\cdot 300^{2}}{4}}{\text{mm}}^{2}=102\text{ MPa}$$

**Figure 2:**
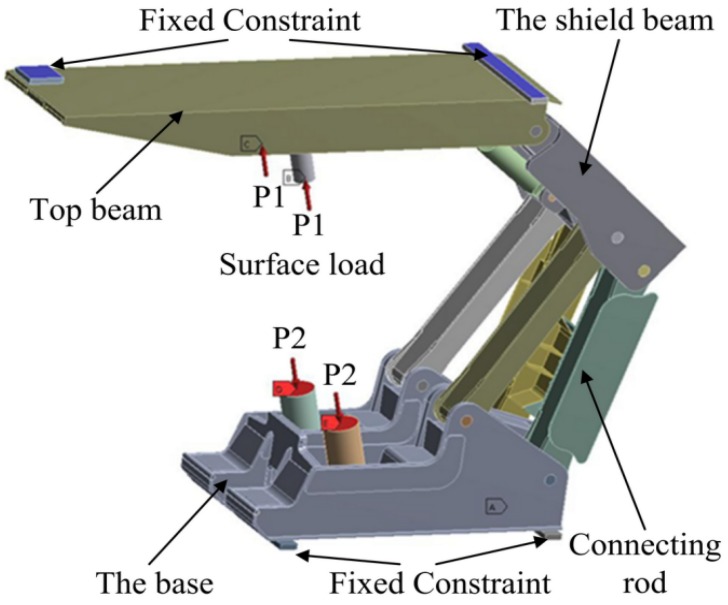
The load and boundary conditions of hydraulic support.

**Figure 3:**
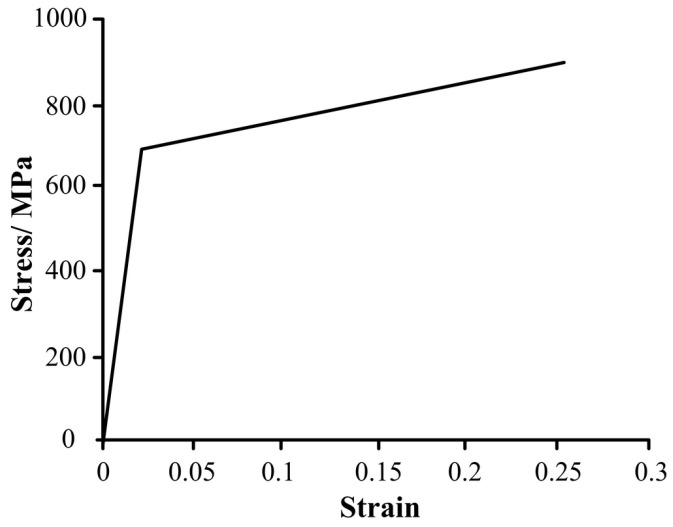
The double linear curve of Q690 steel.

Therefore, the actual working resistance of single pillar is *F*_a_ = 7200 kN. Then, the surface load of live column and outer cylinder rafter converted is 283 and 102 MPa, respectively. The load and boundary conditions of hydraulic support are shown in Figure 2. In this study, the main material of hydraulic support is Q690 steel. In the process of analysis, the double linear constitutive model was adopted for plastic analysis (see Figure 3). The entire geometrical model was meshed with eight-node linear brick elements Soild185.

### 2.2. Fatigue Testing

T-joints are widely used in the connections among main parts (main reinforcement and roof, main reinforcement and cover plate, transverse reinforcement plate and main reinforcement). For the T-joints, fatigue tests are hard to be carried out. Thus, the cruciform welded joint was used to simulate T-joint to carry out fatigue tests. One side of the cruciform welded joint conforms to the characteristics of the T-joint, and the sample can be clamped conveniently in the process of fatigue tests.

Fatigue tests were conducted on 200 kN high frequency fatigue testing machine (CIMACH, Changchun, China) with static load error for full measuring range of ±0.2% and dynamic load error of ±2%. Under tension–tension constant amplitude loading mode, each type of welded joint was tested at five stress levels with stress ratio *R* = 0.1 at room temperature in air environment.

### 2.3. The Design of Welded Joint

Fatigue performance of welded structure mainly depends on the dimensional details of welded joint. In order to improve the fatigue performance of hydraulic support, a better form of welded joint must be designed. The main body of hydraulic support involves welded joints (main reinforcement and roof, main reinforcement and flats, transverse reinforcement plate and main muscle). These welded joints are basic T-type joints. The forms of T-joints can be bilateral symmetry or unilateral symmetry. While designing the welded joint, these two conditions were both covered.

**Figure 4:**
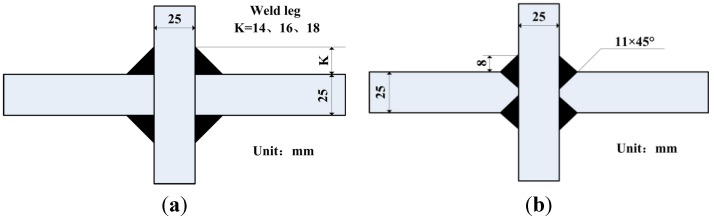
The size and details of bilateral symmetry welded joint. (**a**) Welded joint without groove in *K* = 14, 16 and 18 mm; (**b**) Welded joint of 11 × 45° groove and 8 mm weld leg.

**Figure 5:**
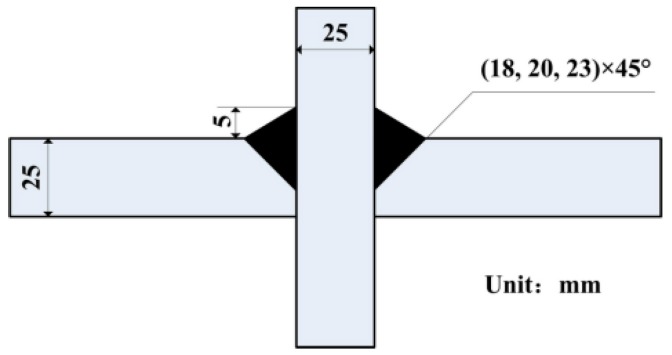
The size and details of unilateral symmetry welded joints.

For the bilateral symmetry welded joint: one was cruciform joint with fillet weld (weld leg size of 14, 16 and 18 mm, respectively); and another was cruciform joint with full penetration weld (weld leg size of 8 mm), which had the nearly same effective sectional area with that of weld leg size of 18 mm (Figure 4). The unilateral symmetry welded joints with different size are schematically shown in Figure 5.

## 3. Results and Discussions

### 3.1. The Stress Field of Finite Element Simulation

**Figure 6:**
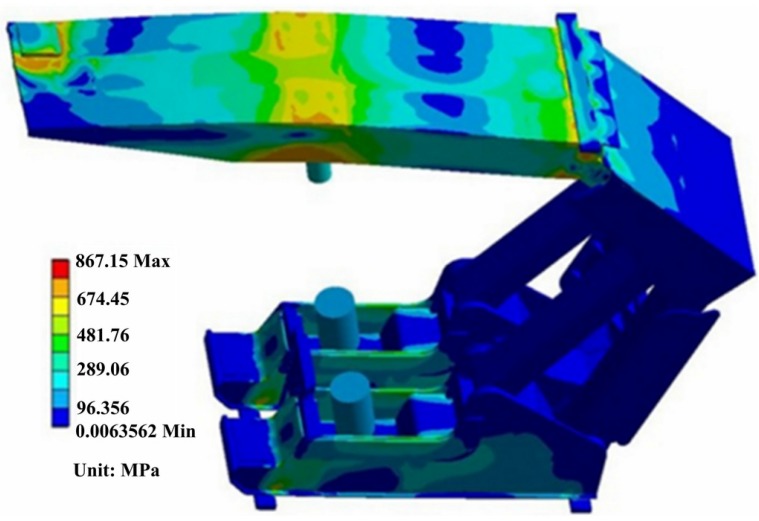
The stress contour of the entire hydraulic support.

The top beam of hydraulic support is welded structure with box shape, which plays the action to support the working surface of the roof in order to prevent gangue and roof coal caving. Figure 6 shows the stress contour of hydraulic support under the condition of the top beam subjected to torsion load and both ends of the base subjected to working load. Stress distribution is very uneven in Figure 6, but most parts present symmetrical distribution. Obviously, the higher stress appears in the top beam and the base. The maximum stress is 867.15 MPa in the top beam.

**Figure 7:**
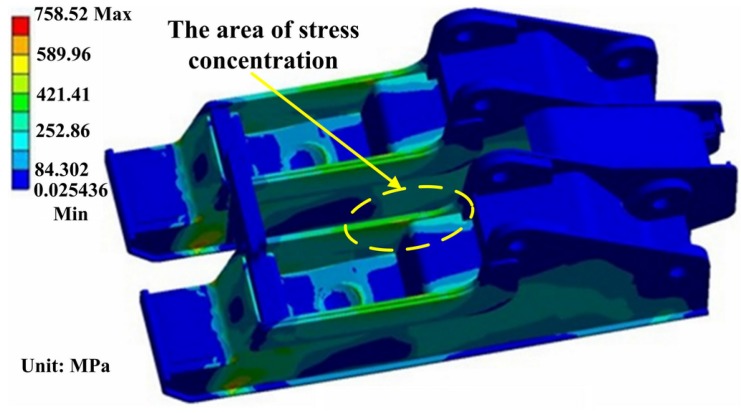
The stress distribution in the base.

The detailed stress distribution of the base is shown in Figure 7.

The stress distribution in both sides of the base is symmetrical with the maximum value of 758.52 MPa. Stress concentration occurs in the border area between bottom plate and main reinforcement or coverplate and main reinforcement. Meanwhile, the stress in the contact zone between the column nest piece and right stiffener plate is also larger.

**Figure 8:**
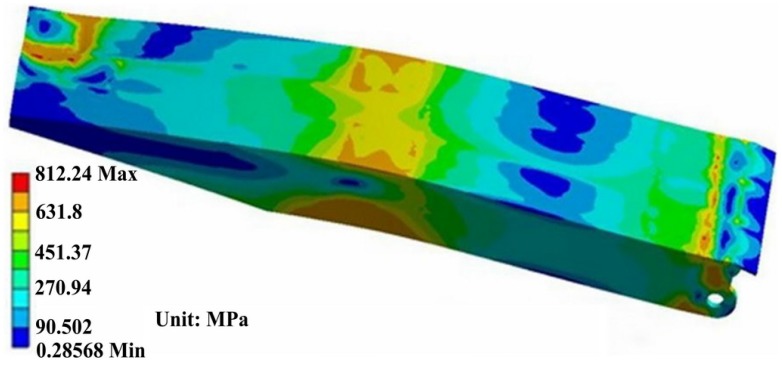
The stress distribution of the front side of the top beam.

**Figure 9:**
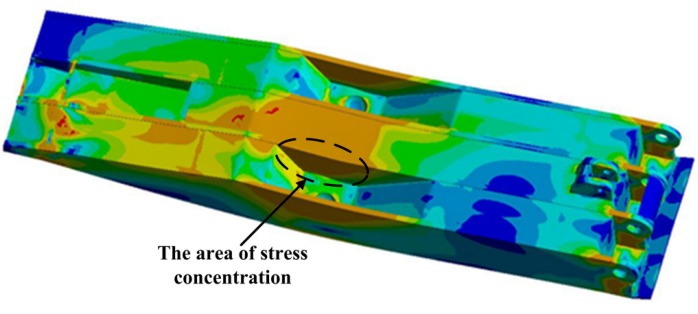
The stress distribution of column nests of the back side of the top beam.

The top beam suffers more severe load under this working condition, as partial load effect is also involved in torsion process. Figure 8 and Figure 9 show the stress distribution of the front and back sides of the top beam, respectively. It can be seen in Figure 8 and Figure 9 that the area of high stress is larger in this working condition. The maximum stress of 812.24 MPa appears near the block. The main stress concentration area mainly distributes in both sides and middle area of the top beam.

**Figure 10:**
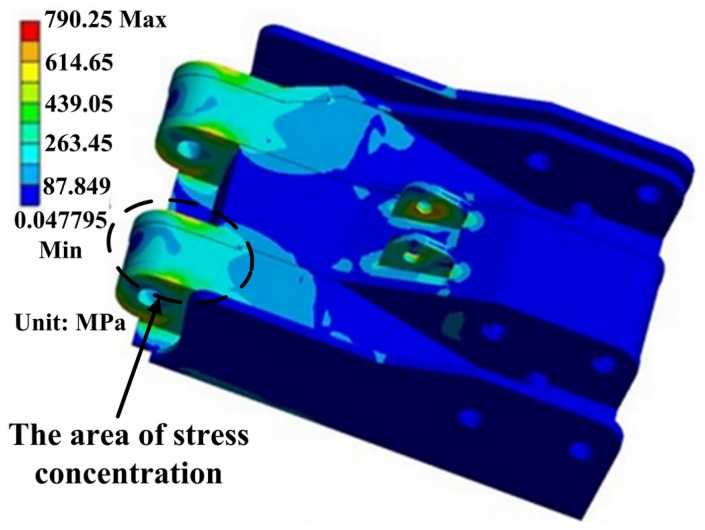
The stress distribution of shield beam.

Figure 10 indicates that the high stress distribution of shield beam. The maximum stress of 790.25 MPa exists in the pin hole connecting to the jack. Meanwhile, the area between coverplate and main stiffened plate also exists stress concentration.

**Figure 11:**
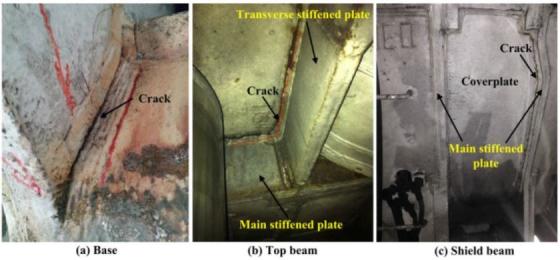
The damaged positions of hydraulic support during actual service.

Simulation results show that the severe stress concentration universally appears in the welded position of the main body. From Figure 7, Figure 9 and Figure 10, it can be seen that the area of stress concentration mainly exists in the base, top beam and shield beam. It is known that the compressive stress can inhibit crack formation and propagation, while the tensile stress is beneficial to crack formation and propagation. For a large welded structure, under the action of external loading, some weld seams bear tensile load, other weld seams bear compressive load. Generally, the weld seam under compressive load will be not easy to crack during service. However, the bearing capacity of weld seam under tensile external load and welded residual tensile stress will be reduced. Figure 11 shows the actual damaged conditions of the base, top beam and shield beam of hydraulic support during actual service. It is observed that the fracture locates at weld toe of the box-shape welded structure, which is consistent with the simulation results. The areas of stress concentration (the actual fracture positions) are indicated by arrow marks in Figure 7, Figure 9 and Figure 10. Therefore, it is important to choose an appropriate form of welded joint to improve the fatigue performance of hydraulic support.

### 3.2. Fatigue S-N Curve

The stress level and fatigue life together determine the fatigue performance of the sample. The relationship between applied stress and fatigue life is represented in Equation (10). The curve based on Equation (10) is the traditional *S*-*N* curve.

(10) $$N=\frac{C}{\text{Δ}{\text{σ}}^{m}}$$

According to *S*-*N* curve, the fatigue strength corresponding to a cyclic number can be obtained. Fatigue testing data should be analyzed according to the principle of statistical method established in IIW (International Institute of Welding) [13] by Hobbacher. The authorized surviving fraction is 50%, while the confidence is 75%.

Nominal value is calculated as the following process:

Calculating all the stress range Δσ and the cycle index *N* (in logarithm with the base-number 10) of fatigue testing data points;

Calculating the exponent *m* and the constant log *C* regressively, using the power function model:

(11) mlogΔσ+logN=logC

**Figure 12:**
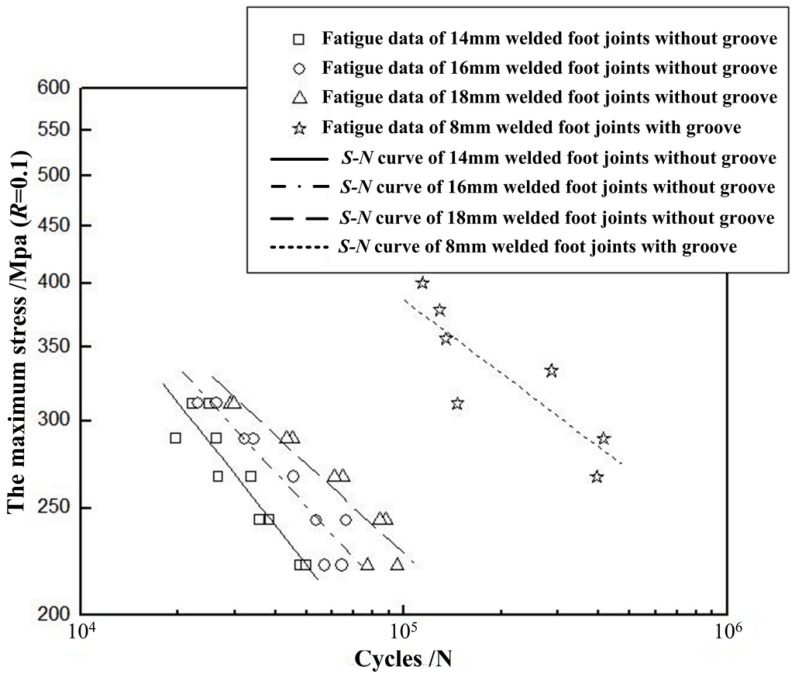
Fatigue *S*-*N* curves of the bilateral symmetry welded joints.

Fatigue *S*-*N* curves of the bilateral symmetry welded joints are shown in Figure 12.

By analyzing *S*-*N* curves in Figure 12, the fatigue performance of joints with different types can be compared. Table 1 presents the maximum stress corresponding to cycle numbers of 30,000, 60,000 and 90,000, respectively. The numbers given in Table 1 are taken from the *S*-*N* curves in Figure 12 obtained by regression analysis.

**Table 1:** Maximum stress corresponding to different cycle numbers.

Cycle Times	Without Groove + 14 mm Weld Leg	Without Groove + 16 mm Weld Leg	Without Groove + 18 mm Weld Leg	Without Groove + 8 mm Weld Leg
30,000	268 MPa	295 MPa	315 MPa	501 MPa
60,000	206 MPa	238 MPa	262 MPa	429 MPa
90,000	179 MPa	208 MPa	234 MPa	393 MPa

It can be observed that fatigue life of the fillet welded joint with bilateral symmetry groove increases with the increment of weld leg size. Compared with that of 16 mm fillet welded joint, fatigue strength of the 18 mm fillet welded joint has been enhanced by 6%, 10% and 12.5% corresponding to cycle numbers of 30,000, 60,000 and 90,000, respectively. For the fully penetrated fillet welded joint with groove of 11 × 45° and weld leg size of 8 mm, compared with that of 18 mm fillet welded joint, fatigue strength has been significantly improved by 59%, 63% and 68% corresponding to cycle number of 30,000, 60,000 and 90,000, respectively. Clearly, the fully penetrated fillet welded joint with groove is extremely advantageous.

**Figure 13:**
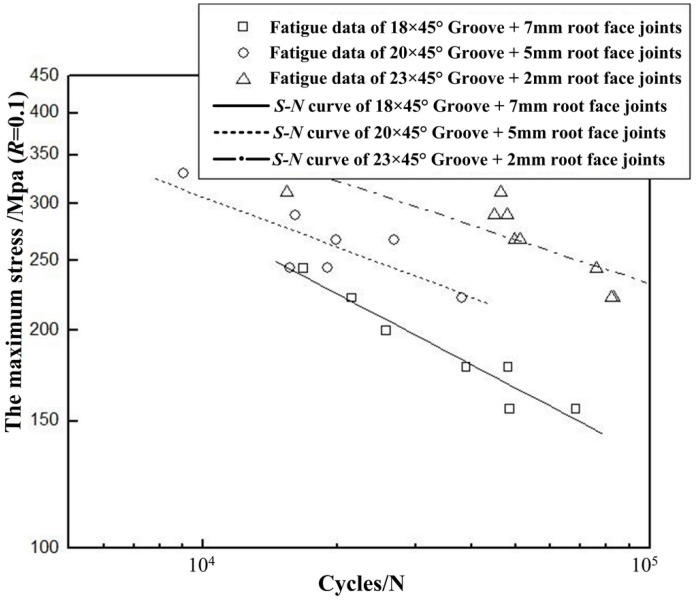
Fatigue *S*-*N* curves of the unilateral symmetry welded joints.

*S*-*N* curves of the unilateral symmetry welded joints with the different size details are shown in Figure 13.

By analyzing the *S*-*N* curves in Figure 13, fatigue performance of the unilateral symmetry welded joints with different size details can be obtained. Table 2 shows the maximum stress corresponding to cycle numbers of 30,000, 60,000 and 90,000, respectively. The numbers given in Table 2 are also taken from the *S-N* curves in Figure 13 obtained by regression analysis.

It can be seen in Figure 13 and Table 2 that fatigue life of the unilateral symmetry welded joint increases as the size of root face decreases. Fatigue strength of the welded joint with groove size of 20 × 45° has been enhanced by 20.4%, 30.4% and 32.4% compared with that with groove size of 18 × 45° corresponding to cycle numbers of 30,000, 60,000 and 90,000, respectively. As for the fully penetrated welded joint with groove size of 23 × 45°, fatigue strength has been improved by 26.3%, 24.8% and 28.3% compared with that with groove size of 20 × 45° corresponding to cycle numbers of 30,000, 60,000 and 90,000, respectively. Therefore, in order to greatly improve fatigue life of the unilateral symmetry welded joint, grooving and full penetration is recommended.

**Table 2:** Maximum stress corresponding to different cycle numbers.

Cycle Number	18 × 45° Groove + 7 mm Root Face Non-Penetration	20 × 45° Groove + 5 mm Root Face Non-Penetration	23 × 45° Groove + 2 mm Root Face Full Penetration
30,000	196 MPa	236 MPa	298 MPa
60,000	158 MPa	206 MPa	257 MPa
90,000	139 MPa	184 MPa	236 MPa

### 3.3. Fatigue Fracture Location

#### 3.3.1. The Bilateral Symmetry Welded Joints

**Figure 14:**
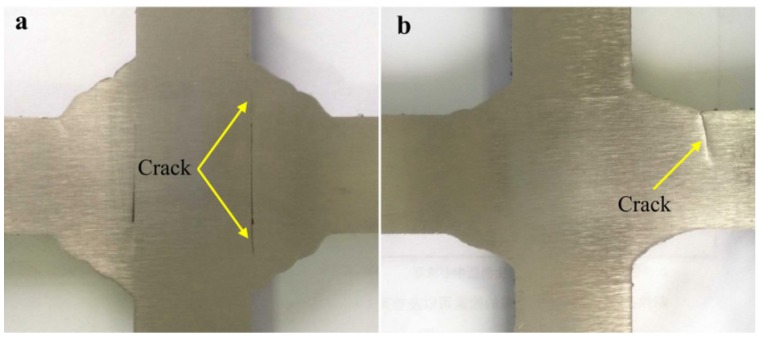
The position of crack initiation for the bilateral symmetry welded joints. (**a**) fatigue fracture is from weld root; (**b**) fatigue fracture is from weld toe.

Figure 14a shows that fatigue fracture is absolutely from the position where weld root was incompletely penetrated.

For the fully penetrated welded joint with groove, fatigue fracture is caused by the stress concentration at weld toe (see Figure 14b). Obviously, by grooving and fully penetrating, fatigue crack initiations all appear at weld toe where stress concentration is relatively serious. Although some defects still exist at weld root (caused by manipulation or groove size), which will not produce fatigue crack. For the bilateral symmetry welded joints without groove, crack initiation usually develops at weld root of the fillet welded joint. However, for the fully penetrated welded joint with groove, fatigue crack initiations all form at weld toe. Fatigue performance cannot be improved through post-processing the weld toe if crack initiates at weld root. Yet, fatigue performance can be enhanced by reducing the stress concentration through grinding or remelting after welding if crack initiates at weld toe. Thus, the fully penetrated joint with groove is recommended for bilateral symmetry weld.

#### 3.3.2. The Unilateral Symmetry Welded Joints

**Figure 15:**
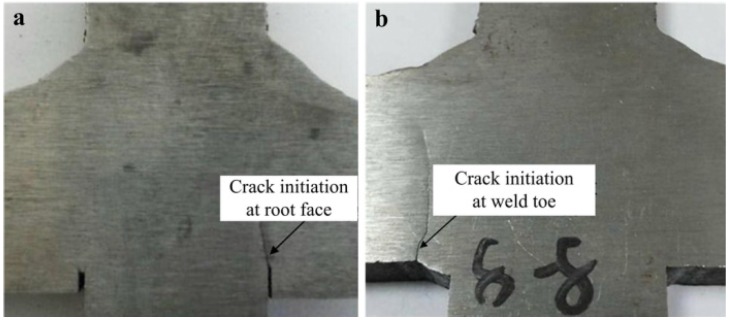
The position of crack initiation for the unilateral symmetry welded joints. (**a**) crack initiation is from root face; (**b**) crack initiation is from weld toe.

Fatigue cracks of the incompletely penetrated joints with groove size of 18 × 45° and root face of 7 mm as well as the incompletely penetrated joints with groove size of 20 × 45° and root face of 5 mm are all caused by the natural stress concentration at root face (see Figure 15a).

For the fully penetrated joints with groove size of 23 × 45° and root face of 2 mm, the root face of 2 mm has not existed after welding. Thus, the position of fatigue fracture will change from weld face into weld toe owing to the fully penetrated root face (see Figure 15b).

### 3.4. The Analysis of Conventional Fatigue Fracture Mechanism

**Figure 16:**
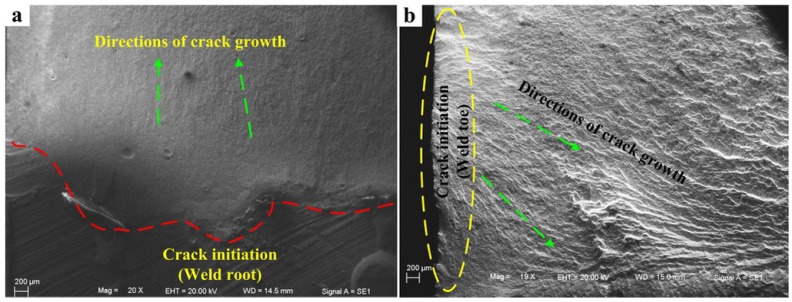
Crack initiation for the bilateral symmetry welded joints. (**a**) crack initiation is from weld root; (**b**) crack initiation is from weld toe.

For the bilateral symmetry welded joints, fatigue initiations of the joints without groove and the joints with groove are at weld root (Figure 16a) and weld toe (Figure 16b), respectively. The specific characteristics of crack initiation are shown in Figure 16.

**Figure 17:**
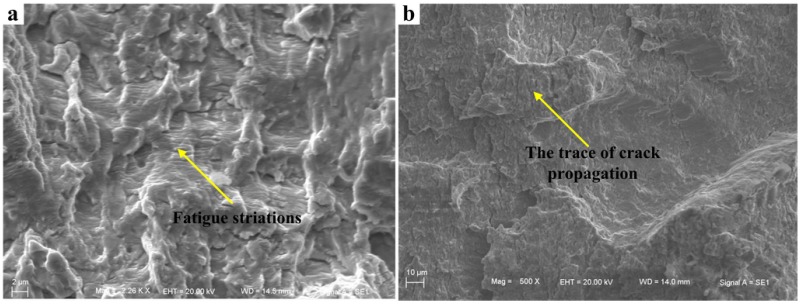
Fatigue striations in crack propagation region of the bilateral symmetry welded joints. (**a**) crack propagation region corresponding to Figure 16a; (**b**) crack propagation region corresponding to Figure 16b.

Figure 17a indicates that the obvious fatigue striations have been generated for the defects caused by weld root. The reason is that the force along the direction of crack propagation at weld root is relatively small compared to that at weld toe. On the contrary, due to the larger stress at weld toe, crack extension rate under this state is relatively fast. Thus, fatigue striations are not obvious. Only the trace of crack propagation can be observed in the low-magnification Microscope (Carl Zeiss Jena, Oberkochen, Germany) (see Figure 17b).

**Figure 18:**
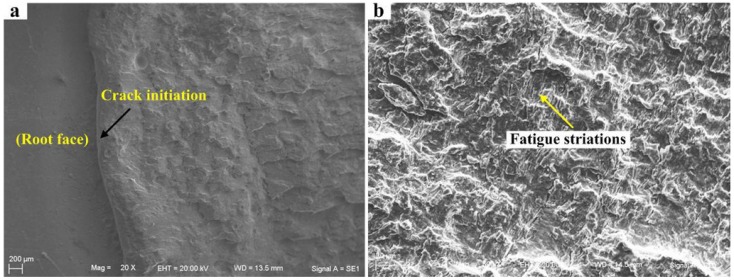
Fatigue crack initiation and fatigue striations of the incompletely penetrated joints for the unilateral symmetry welded joints. (**a**) crack initiation; (**b**) fatigue striation.

For the unilateral symmetry welded joints, with the decrease of the size of root face, penetration performance and fatigue resistance of joints have been significantly improved. The position of fatigue initiation also shifts from weld root to weld toe (see Figure 15b). Therefore, fatigue fracture position of the penetrated joint is at weld toe, and its fatigue crack initiation morphology is consistent with that in Figure 16b. Fatigue crack initiation region and fatigue crack propagation region of the incompletely penetrated joints are shown in Figure 18.

**Figure 19:**
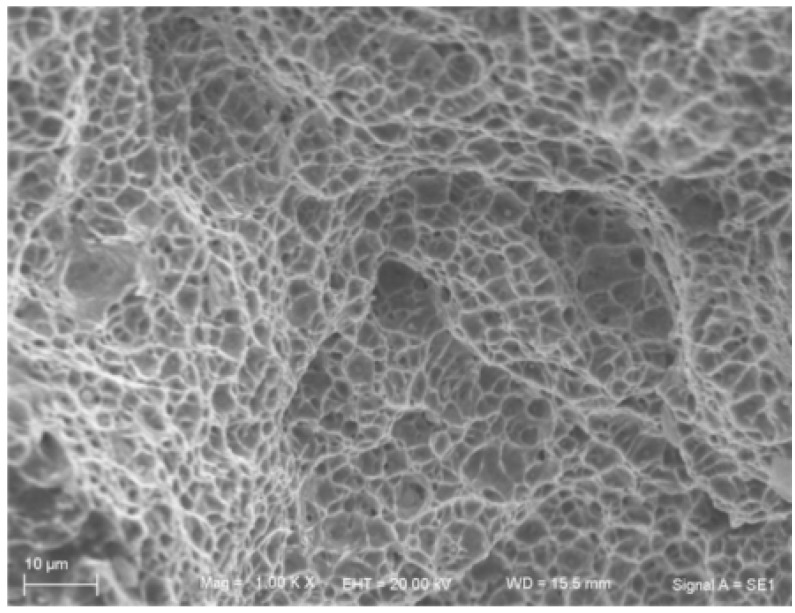
Fracture section of the instantaneous destruction region.

Fracture section of the instantaneous destruction region has the typical characteristic of dimple, which indicates that the fracture belongs to ductile fracture. In particular, the second phase particles still exist in the dimples, showing excellent ductility characteristics (see Figure 19).

## 4. Conclusions

By analyzing the structural characteristics of hydraulic supports based on simulation and fatigue testing, we can draw the following conclusions:

(1) Simulation results are consistent with the actual damaged positions of hydraulic supports.
(2) Structural stress concentration is the main factor leading to the fatigue damage of hydraulic supports. The key point of fatigue design of welded structure is to avoid or reduce stress concentration.
(3) This research provides a theoretical reference and a certain guiding significance for the fatigue design of hydraulic supports.
